# Seroprevalence of *Mycobacterium lepraeantibodies* among school children in Indonesia in 2023: a cross-sectional study

**DOI:** 10.1016/j.lansea.2026.100779

**Published:** 2026-05-27

**Authors:** Louise Pierneef, Munawir Muhammad, Gaby Wassenaar, Daniel Edbert, Hana Krismawati, Sitti Wahyuni, Soegianto Ali, Maria Mardalena Martini Kaisar, Paul Corstjens, Annemieke Geluk

**Affiliations:** a Leiden University Center for Infectious Diseases, Leiden University Medical Center, Leiden, the Netherlands; b Department of Pharmacology, Faculty of Medicine, Hasanuddin University, Makassar, Indonesia; c Department of Microbiology, Faculty of Medicine, School of Medicine and Health Sciences, Atma Jaya Catholic University of Indonesia, Jakarta, Indonesia; d Center of Health System and Strategy, Ministry of Health, Jakarta, Indonesia; e Department of Parasitology, Faculty of Medicine, Hasanuddin University, Makassar, Indonesia; f Master in Biomedicine Study Program, School of Medicine and Health Sciences, Atma Jaya Catholic University of Indonesia, Jakarta, Indonesia; g Department of Parasitology, School of Medicine and Health Sciences, Atma Jaya Catholic University of Indonesia, Jakarta, Indonesia; h Department of Cell and Chemical Biology, Leiden University Medical Center, Leiden, the Netherlands

**Keywords:** Children, Helminths, *M. leprae* infection, PGL-I QURapid, Rural vs. urban, Transmission

## Abstract

**Background:**

Leprosy remains a significant public health issue in endemic regions, including areas in Indonesia. Transmission of *Mycobacterium leprae (M. leprae)* continues as clear from the almost stable annual number of new leprosy cases amongst which around 5% are children. To interrupt transmission of *M. leprae*, it is essential to identify and treat infection sources. This study aimed to assess anti-*M. leprae* phenolic glycolipid-I (PGL-I) IgM seroprevalence among school-aged children in rural and urban Indonesia, as a proxy for recent transmission. Additionally, associations between seropositivity and gender, socioeconomic status (SES), and helminth infection were investigated.

**Methods:**

We conducted a cross-sectional serosurvey among 637 children (ages 6–15) from schools in urban and rural areas of Sulawesi, Java, and Sumba, Indonesia. The PGL-I QURapid, a field-friendly and quantitative lateral flow assay, was used to detect anti-PGL-I IgM in fingerstick blood and plasma samples. Socioeconomic and health data, including helminth infection status and body-mass index z-scores (z-BMI), were also collected.

**Findings:**

The overall seroprevalence of anti-PGL-I IgM in children was 12.2% with significantly higher seroprevalence in rural (Southwest Sumba: 31.0%, Pangkajene: 11.2%) compared to urban areas (North Jakarta: 3.8%, Makassar city: 3.1%). Moreover, seroprevalence amongst children from low-SES schools was significantly higher (13.7%) than among those from high-SES schools (4.1%, *P* = 0.007). The presence of helminth infections were associated with significantly higher anti-PGL-I IgM levels (*P* < 0.0001), with 22.4% of helminth-infected children testing seropositive compared to 8.8% of non-infected children (*P* < 0.001).

**Interpretation:**

Our findings demonstrate a high anti-PGL-I IgM seroprevalence among rural, low-SES, and helminth-infected children in Indonesia, indicating elevated levels of *M. leprae* infection within these groups which again represents increased transmission rates in the population. These results underscore the need for integrated disease control strategies addressing both leprosy and helminthiasis, particularly in rural and economically disadvantaged areas. The PGL-I QURapid demonstrated to be an effective tool in serosurveys, able to aid the identification of areas with high *M. leprae* transmission rates (such as Sumba) and thereby support the implementation of targeted interventions in endemic regions.

**Funding:**

This study was funded by a grant from the 10.13039/501100009425Q.M. Gastmann-Wichers Foundation (AG) and an internal grant from 10.13039/501100019588Atma Jaya Catholic University of Indonesia (MMMK).

**Research in context**

**Evidence before this study**

Leprosy remains a major public health challenge in several endemic regions, including Indonesia, despite the availability of curative multidrug therapy. The WHO aims to achieve “zero leprosy” by 2030. This includes zero leprosy infection and disease, zero disability, and zero stigma and discrimination. To monitor whether leprosy is eliminated in an area, serological detection of anti-phenolic glycolipid-I (PGL-I) IgM antibodies in young children has been recognized by the WHO as a proxy for recent *Mycobacterium leprae* transmission. However, large-scale, child serosurveys in diverse socioeconomic and geographic settings are lacking.

**Added value of this study**

This is the first multi-site study in Indonesia to assess anti-*M. leprae* PGL-I IgM seroprevalence among school-aged children across urban and rural regions in Indonesia using the PGL-I QURapid - a robust, low-complexity, and quantitative lateral flow assay. Moreover, associations between *M. leprae* infection and socioeconomic status (SES), nutrition, and helminth co-infections were assessed. The study identified an overall anti-*M. leprae* seroprevalence of 12.2% in children, with significantly higher rates in rural and low-SES settings (up to 31%). Further it demonstrates that among helminth-infected children, seropositivity was significantly higher (22.4%) compared to non-infected children (8.8%). The study also confirms the operational feasibility of the PGL-I QURapid in serosurveys within resource-limited settings to monitor recent *M. leprae* transmission.

**Implications of all the available evidence**

Our data indicate that *M. leprae* transmission persists in these parts of Indonesia, particularly in rural and socioeconomically disadvantaged communities and that differences can be detected. The findings underscore the need for integrated disease control strategies tailored to high-risk populations. Furthermore, serological screening of school-aged children can serve as a valuable tool for identifying priority regions for leprosy post-exposure prophylaxis and active case finding. Incorporating anti-*M. leprae* PGL-I serosurveillance - using a field-friendly diagnostic test - into national leprosy control programs could significantly improve early detection and support in monitoring of intervention efficacy.

## Introduction

Leprosy, caused by *M. leprae (M. leprae)* or *Mycobacterium lepromatosis (M. lepromatosis)*, is a neglected tropical disease that primarily affects the skin, peripheral nerves, upper respiratory tract, and eyes.^1^ Although curable with multidrug therapy (MDT), leprosy remains a significant public health concern, particularly in low-income countries where healthcare access is limited, such as Indonesia.^2^ Despite ongoing control efforts, many individuals continue to suffer from the long-term consequences of leprosy, including physical disability and social stigma.

A majority of exposed individuals do not develop clinical disease as they are adequately protected by their immune system,^3^ but asymptomatic carriers can still harbor the bacteria in the nasopharyngeal cavity and could contribute to transmission,^4^ complicating control strategies. Notably, the incubation period can generally span 2–10 years, but even over 50 years has been reported.^5^ Close and prolonged contact with untreated leprosy patients remains a primary risk factor, placing household contacts at the highest risk of developing the disease.^6^ Other factors reported to be associated with increased risk of leprosy are low socioeconomic status (SES), malnutrition, and substandard living conditions.^7^ Moreover, in leprosy endemic areas, coinfection with soil-transmitted helminths (STH) is common.^8^ Pre-existing helminth infections may enhance susceptibility to *M. leprae* infection or exacerbate disease severity through modulation of host immune responses.^8^^,^^9^ Given the high burden of helminths in leprosy endemic regions, integrated disease control strategies are vital.

To successfully interrupt transmission of *M. leprae*, it is essential to identify and treat infection sources early. Currently, leprosy elimination efforts are monitored by assessing the proportion of new cases in children under 15 years of age among the total number of newly detected cases. However, since only a small fraction of individuals infected with *M. leprae* develop clinical disease - and because symptoms may take years to appear - this indicator does not provide timely or sufficiently accurate insight into ongoing transmission. Instead, monitoring infection rates could offer a more informative and responsive measure to guide intervention strategies, assess transmission trends, and track progress toward elimination.^10^^,^^11^ Due to its usefulness in detecting infection among asymptomatic individuals,^12^, ^13^, ^14^ the WHO recommends using *M. leprae*-specific anti-phenolic glycolipid-I (PGL-I) serology in children as a tool for assessing ongoing *M. leprae* transmission. PGL-I is a glycolipid found exclusively in the cell wall of *M. leprae and M. lepromatosis*, highlighting its specificity for the leprosy causing pathogens. IgM levels against this antigen correlate strongly with the bacterial load in infected individuals,^15^ making it a useful marker for the identification of those most likely to contribute to transmission. Anti-PGL-I IgM can be present in individuals with either past or present *M. leprae* infection. As infection in (young) children typically indicates recent transmission (since it cannot exceed their age), the detection of antibodies in this group likely indicates recent or ongoing infection, making them a preferred group to monitor seroprevalence and thus transmission.

The type of serological test and standardisation of the cut-off for positivity used in seroscreening for assessment of transmission is crucial.^12^ To facilitate field-based surveillance, we have developed the PGL-I QURapid; a low-cost, user-friendly lateral flow assay employing upconverting reporter particle (UCP) technology for the quantitative detection of anti-PGL-I IgM in capillary or venous blood.^16^ This test provides a practical and scalable tool for resource-limited settings, offering application in multiple use cases globally.

In 2000, the WHO announced that Indonesia had effectively eliminated leprosy,^17^ meaning that prevalence had fallen below the WHO-defined epidemiological threshold, which is distinct from complete elimination of the disease from the population. This led to significant downscaling of the national leprosy control program, especially affecting areas with limited medical services. Still, Indonesia ranks as the third-highest contributor to the global leprosy burden, with approximately 12,000–17,000 new cases reported annually.

In this study, we assessed the seroprevalence of anti-PGL-I IgM among school-aged children from rural and urban areas across Indonesia using the PGL-I QURapid. By exploring associations between seropositivity and factors such as gender, SES, and helminth infection, we sought to identify environmental and host-related determinants of *M. leprae* infection. The resulting insights may inform public health strategies for leprosy control and support the implementation of targeted interventions by identifying hotspots for *M. leprae* transmission.

## Methods

Study participants were schoolchildren (n = 637; age 6–15 years) from different socioeconomic backgrounds living in rural and urban parts of three areas in Indonesia (Supplementary Fig. S1).

### Sulawesi

321 children from two elementary schools located in the urban centre of Makassar (Makassar city District), as well as two elementary schools situated on the rural island of Karanrang (Pangkajene dan Kepulauan District). Sample and data collection took place between September and November 2023. Schools differed in socioeconomic status (SES); three schools were designated “low-SES” and one school “high-SES”.^18^ The high-SES school was located in the city center and is considered of high-SES status, with a majority of the parents working as high-skilled workers or professionals with higher education. Meanwhile, the low-SES schools were located near landfill and port areas, where most of the parents are low-educated and work in low-skilled labor jobs.

### Java

158 children from (low-SES) public schools in the Kamal Muara (KM) sub-district, located within the Penjaringan District of North Jakarta, an urban-slum area in Java.

### Sumba

158 children from the rural (low-SES) Hameli-Ate sub-district within the North Kodi District of Southwest Sumba, located in eastern Indonesia.

Within each district, schools were purposively selected based on existing collaborations, prior contact with the research team and approval by the headmasters, as part of a bigger study. Schools were chosen to represent both urban and rural settings and, where possible, different socioeconomic backgrounds. Children from all classes in the selected schools who were willing to participate and for whom parental informed consent was obtained were invited to take part. The study sites on Sumba and Java aimed for approximately similar numbers of participants at each site (around 160 children). For the other sites (South Sulawesi), numbers were dependent on the number of schoolchildren available at the schools focused on in this sub-study, and set to allow for meaningful comparisons across all study sites.

New case detection rates (NCDRs) by district for 2023 are presented in Supplementary Table S1.^19^

Information regarding demographics and indicators of SES was obtained using a questionnaire. Body weight and height were measured and used to calculate the age-standardised z-scores of body mass index (z-BMI), according to WHO guidelines. For the purpose of this study, the categorisation of BMI-for-age was simplified by classifying all children with a z-score lower than −2 as “underweight”, all children with a z-score between −2 and 1 as “normal weight” and all children with z-scores above 1 as “overweight”. BCG vaccination was assessed by examining the presence of a BCG scar on the upper arm, measured as the mean of two diameters using a ruler.

#### Parasitological examination

Same day stool specimens were obtained from each child and subjected to microscopic examination upon arrival at the laboratory. Stool specimens were examined using Kato-Katz technique in accordance with WHO recommendations for the detection of STH, such as *Ascaris lumbricoides*, *Trichuris trichiura* and hookworm.^20^ For stool specimens collected from the KM area (Java), additional microscopy techniques were employed: a direct smear and Haradamori (a culture-based microscopy technique) targeting the larvae of hookworms or *Strogyloides stercoralis*.^21^

#### Fingerstick blood collection

FSB was collected using disposable 20 μl Minivette ® collection tubes (Heparin coated; Sarstedt) and directly mixed with 980 μl high salt finger stick (HSFS) buffer: 100 mM Tris pH 8.0, 270 mM NaCl, 1% (v/v) Triton X-100, and 1% (w/v) BSA providing a 50-fold dilution.^13^^,^^16^

#### Plasma specimen collection

The whole blood samples were collected from KM and HA areas, in 0.5 ml EDTA vacutainer tubes (BD, Franklin Lakes, NJ, USA). Tubes were centrifuged at 2500 rpm for 10 min and plasma was subsequently aliquoted and frozen (−80 °C) until further use.

#### PGL-I QURapid

Individually packaged UCP-LFA cassettes for the detection of human anti-PGL-I IgM antibodies were produced by Maxim Biomedical Inc. (Rockville, MD, USA) as described previously.^13^ The air-tight pouches with test cassettes contained silica dry packs for extended shelf life and protection against humidity. The Test (T) line on the LF strip (nitrocellulose membrane; Sartorius UniSart CN95) comprised 100 ng of synthetic PGL-I, phenolic trisaccharide functionalized with a hexanoic acid linker for conjugation to BSA (NPT1-H-BSA; Leiden, the Netherlands).^22^ The flow-control (FC) line comprised 100 ng rabbit anti-goat IgG (G4018; Sigma–Aldrich, Inc., St. Louis, MO, USA). Goat IgG against human IgM (I0759; Sigma–Aldrich, Inc., St. Louis, MO, USA) was conjugated to polyacrylic acid functionalized UCPs [200 nm, NaYF_4_:Yb^3+^, Er^3+^; Intelligent Material Solutions Inc. (IMS); Princeton, NJ, USA] according to previously described protocols at a concentration of 50 μg antibody per mg UCP.^23^ To dry the UCPs onto the glass fiber conjugate-release pad, the material was diluted in a buffer containing 100 mM Tris pH 8.0, 270 mM NaCl, 10% (w/v) sucrose, 1% (w/v) BSA, 0.5% Tween-20, and striped at a density of 100 ng/mm.

#### UCP-LFA

50 μl of a 50-fold diluted FSB- or plasma sample was added to the LF strip to initiate LF. PGL-I QURapid-tests were analyzed using a battery-operated UCP-adapted portable lightweight standalone reader (ESEQuant LFR adapted for UCP; DIALUNOX, Stockach, Germany).^24^ Results were calculated as the Ratio (R-)value between the test line (T) and the flow control line (FC) signal based on relative fluorescence units (RFUs) measured at the respective lines. A quality control (QC) protocol was designed to monitor test reproducibility using sera of clinically diagnosed leprosy patients selected based on their anti-PGL-I IgM levels in standard anti-PGL-I IgM ELISAs^15^^,^^22^: a highly seropositive, a seropositive with an OD indicative for the cut-off for seropositivity with the corresponding anti-PGL-I IgM ELISAs (QC reference) and a seronegative serum sample (from a healthy Dutch blood bank donor without travel history to leprosy endemic areas). For direct comparison of FSB and plasma test data, results were normalized and presented as anti-PGL-I ‘units’. A seropositivity cut-off of ≥0.16 was applied for serum/plasma, and ≥0.12 for FSB. The determination of these cut-offs was described previously.^16^ In the reported study, the WHO TPP published in 2023^25^ was leading in terms of required minimum Sn/Sp for establishing a cut-off. Assuming a linear relation between R-value and concentration above the cut-off value, units were calculated by dividing individual R-values by the respective cut-off value, in analogy to methods used in anti-PGL-I ELISAs.^26^^,^^27^ Units ≥1 were considered seropositive.

#### Statistical analysis

This study was conducted in four purposively selected districts and a limited number of schools within each district. Data clustering by school was not accounted for in the analyses. Consequently, all analyses should be considered exploratory, aimed at describing patterns rather than generating nationally representative estimates. The statistical software GraphPad Prism version 9.0.1 for Windows (GraphPad Software, San Diego, CA, USA) and IBM SPSS Statistics for Windows version 29 (IBM Corp, Armonk, NY, USA) were used to perform statistical analysis. Mann–Whitney U and Kruskal–Wallis tests were used to assess differences between two and three independent groups, respectively. Differences in seroprevalence between groups in cross-sectional analyses were evaluated using Chi-squared tests. When information regarding age, gender, helminth infection status, or z-BMI was missing, those individuals were excluded only from the analyses requiring that specific variable, but they remained included in all other analyses for which their data were complete.

#### Ethics statement

This study used samples collected during two population studies conducted previously^21^[Ali S. et al., manuscript under review]. The use of these samples in the current serosurvey was approved by the Ethical Committee of the School of Medicine and Health Sciences (SMHS), AJCUI (Ref. No.: 0004F/III/PPPR.PM.10.05/04/2024). The study in Sulawesi was approved by the Hasanuddin University Ethical Committee (Ref. No.: UH23080619.01/11/2023). Parents were informed about the study and written parental consent was obtained for each child to participate in the study prior to sample collection.

#### Role of the funding source

The funder of the study had no role in the study design, data collection, data analysis, data interpretation, or writing of the report, and had no influence on the decision to submit the paper for publication.

## Results

### Study participants

To assess *M. leprae* transmission in rural and urban areas with varying SES in Indonesia, we measured anti-PGL-I IgM seroprevalence using the PGL-I QURapid among 637 school-aged children from elementary schools located in North Jakarta, Makassar city, Pangkajene and Southwest Sumba. Of the participants, 51.2% were female, with a median age of 9 years (range: 6–15; Table 1). The majority (84.6%) attended low-SES schools, while 15.4% attended high-SES schools. Half of the children (50.1%) were from rural areas. Helminth infections were present in 28.3% of the participants, 20.1% were classified as underweight and 18.1% as overweight.

**Table 1:** Characteristics of study participants.

Characteristic	Total population N = 637
Age (in years, median, range)^a^	9 (6–15)
Sex (female %, n/N)^b^	51.2 (325/635)
School SES (%, n/N)	
Low-SES	84.6 (539/637)
A	15.4 (98/637)
B	9.9 (63/637)
C	9.7 (62/637)
D	24.8 (158/637)
E	24.8 (158/637)
High-SES	15.4 (98/637)
Location (%, n/N)	
Rural	50.1 (319/637)
Pangkajene	25.3 (161/637)
Southwest Sumba	24.8 (158/637)
Urban	49.9 (318/637)
Makassar city	25.1 (160/637)
North Jakarta	24.8 (158/637)
Helminth infection (%, n/N)^c^	28.3 (170/601)
Nutritional status (%, n/N)^d^	
Underweight	20.1 (116/576)
Normal weight	61.8 (356/576)
Overweight	18.1 (104/576)
All	637

The number of positives (n) of the total number (N). Low-SES A-E refer to different low-SES schools included in the study.

a Age info was missing for 12 participants.

b Gender info was missing for two participants.

c Helminth infection status info was missing for 36 participants.

d z-BMI scores were used to classify weight groups. z-BMI < −2: underweight; z-BMI ≥ −2 and ≤1: normal weight; z-BMI >1: overweight. z-BMI scores were missing for 61 participants.

### Seroprevalence, gender differences and BCG

Among the 637 children tested, 78 (12.2%) were seropositive for anti-PGL-I IgM (Table 2). Female children had significantly higher levels of anti-PGL-I IgM compared to male children (Supplementary Fig. S2A; Mann–Whitney: *P* < 0.0001). Additionally, seropositivity rates were significantly higher in females (n = 50, 15.4%) than in males (n = 28, 9.0%; Chi-squared: *P* = 0.016; Supplementary Fig. S2B). No significant age-related differences in anti-PGL-I levels were observed (Supplementary Fig. S2C). Furthermore, BCG-scar size did not appear to influence seropositivity quantitatively as there was no correlation between size of the scar and levels of anti-PGL-I IgM (data not shown). The distribution of anti-PGL-I R-values is shown in Supplementary Fig. S3.

**Table 2:** Anti-PGL-I IgM seropositivity per study area.

Island	Province	District	Positive [n, (%)]	Total (n)
Java				
	Special Capital Region of Jakarta	North Jakarta (U)	6 (3.8)	158
Sulawesi				
	South Sulawesi			
		Makassar city (U)	5 (3.1)	160
		Pangkajene (R)	18 (11.2)	161
Sumba				
	East Nusa Tenggara			
		Southwest Sumba (R)	49 (31.0)	158
All			78 (12.2)	637

U = Urban; R = Rural.

### Rural vs. urban areas

**Fig. 1:**
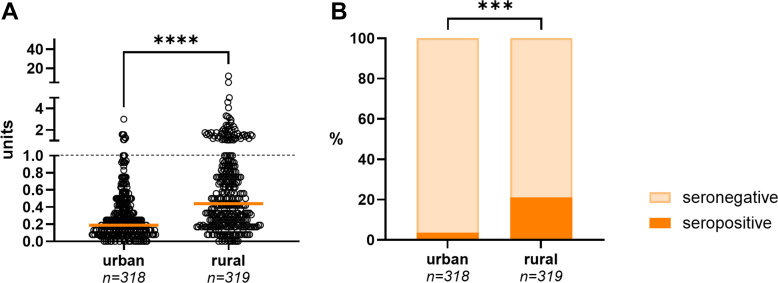
**Anti-PGL-I IgM in children in urban and rural areas across Indonesia. A**: Anti-PGL-I IgM (in units; *y*-axis) of children from urban and rural areas (*x*-axis). Dots (open circles) represent anti-PGL-I units for individual samples. A Mann–Whitney U test was performed to determine differences between the two groups (∗∗∗∗*P* ≤ 0.0001). Anti-PGL-I units ≥1 (dotted line) were considered seropositive. **B**: Percentages (%) of children testing seropositive (*y*-axis) for anti-PGL-I IgM stratified by urban and rural area (*x*-axis). Chi-squared tests were performed to test for differences in percentages between the two groups (∗∗∗*P* ≤ 0.001). IgM: immunoglobulin M; PGL-I: phenolic glycolipid-I.

Seroprevalence was lowest in urban areas, with Makassar city (3.1%) and North Jakarta (3.8%) showing the smallest proportions, while notably higher rates were observed in rural areas: Southwest Sumba (31.0%) and Pangkajene (11.2%; Table 2) (Fig. 1).

**Fig. 2:**
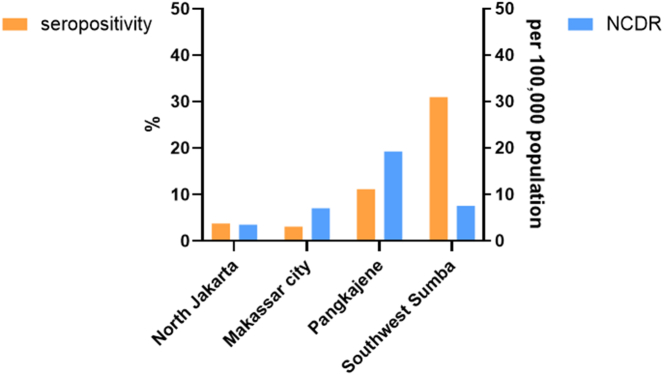
**Anti-PGL-I IgM seropositivity in children, stratified by study district and compared with the corresponding leprosy new case detection rate (NCDR).** Seropositivity percentages are shown in orange (left *y*-axis) for North Jakarta, Makassar City, Pangkajene dan Kepulauan, and Southwest Sumba (*x*-axis). NCDR per district is shown in blue (right *y*-axis).

When comparing seroprevalence in healthy young children with the leprosy patients’ NCDR in the same area, we observed that the relatively lower NCDR in North Jakarta (3.5/100,000) and Makassar city (7.1/100,000) corresponded with lower seroprevalence (3.1–3.8%) of anti-PGL-I IgM in healthy young children, whereas the higher NCDR in Pangkajene (19.3/100,000) corresponded with higher seroprevalence (11.2%; Fig. 2).

### High-SES vs. low-SES

**Fig. 3:**
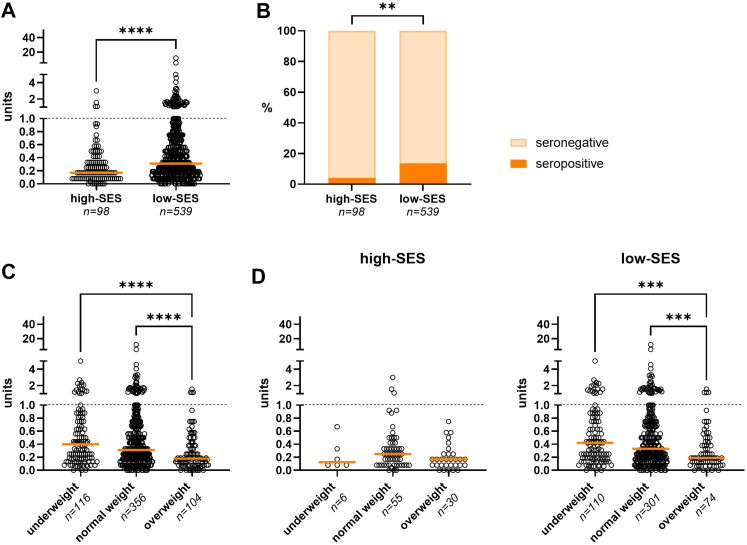
**Anti-PGL-I IgM in children attending high-SES and low-SES schools in Indonesia. A**: Anti-PGL-I IgM (in units; *y*-axis) of children attending high-SES and low-SES schools (*x*-axis). Dots (open circles) represent anti-PGL-I units for individual samples. A Mann–Whitney U test was performed to determine differences between the two groups (∗∗∗∗*P* ≤ 0.0001). **B**: Percentages (%) of children seropositive (*y*-axis) for anti-PGL-I IgM stratified for SES; high and low (*x*-axis). Anti-PGL-I units ≥1 (dotted line) were considered seropositive. Chi-squared tests were performed to test for differences in percentages between the two groups (∗∗*P* ≤ 0.01). IgM: immunoglobulin M; PGL-I: phenolic glycolipid-I. **C**: Anti-PGL-I IgM (in units; *y*-axis) of children classified as being underweight, having normal weight or being overweight (*x*-axis). PGL-I units ≥1 (dotted line) were considered seropositive. A Kruskal–Wallis test with Dunn’s correction for multiple testing was performed to determine differences between the three groups (∗∗∗∗*P* ≤ 0.0001). z-BMI scores were used to classify weight groups. z-BMI < −2: underweight; z-BMI ≥ −2 and ≤1: normal weight; z-BMI >1: overweight. z-BMI scores were not recorded for 61 participants. **D**: Anti-PGL-I IgM levels (*y*-axis) stratified by SES (left panel: high-SES; right panel: low-SES) for children classified as underweight, normal weight, or overweight (*x*-axis).

Given that leprosy is often associated with povertyas well as malnutrition, we examined *M. leprae* infection rates between children differing in z-BMI scores attending high-SES and low-SES schools. The data showed that children from low-SES schools had significantly higher anti-PGL-I levels than those from high-SES schools (Mann–Whitney: *P* < 0.0001; Fig. 3A). A significantly higher percentage (13.7%) of children from low-SES schools tested seropositive for anti-PGL-I IgM (high-SES: 4.1%; Fig. 3B; Chi-squared: *P* = 0.007). Anti-PGL-I IgM levels were significantly higher in children who were underweight or of normal weight compared to those who were overweight (Fig. 3C; Kruskal–Wallis: both *P* < 0.0001). After stratifying by SES, the association persisted only in the low-SES group (Fig. 3D).

### Helminth infection

Helminth positivity was substantially higher in rural areas (Pangkajene: 42.6%; Southwest Sumba: 57.0%) in comparison to urban areas (Makassar city: 14.8%; North Jakarta: 0%; Table 3).

**Table 3:** Helminth positivity per study area.

Island	Province	District	Positive [n, (%)]	Total (n)
Java				
	Special Capital Region of Jakarta	North Jakarta (U)	0 (0)	158
Sulawesi				
	South Sulawesi			
		Makassar city (U)	22 (14.8)	149
		Pangkajene (R)	58 (42.6)	136
Sumba	East Nusa Tenggara	Southwest Sumba (R)	90 (57.0)	158
All			170 (28.3)	601^a^

U = Urban; R = Rural.

a Helminth infection status info was not recorded for 36 participants.

**Fig. 4:**
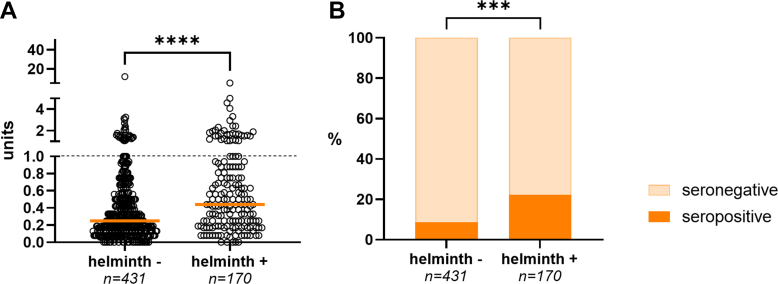
**Anti-PGL-I IgM in children with and without helminth infection in Indonesia. A**: Anti-PGL-I IgM (in units; *y*-axis) of children with and without helminth infection (*x*-axis). Dots (open circles) represent anti-PGL-I units for individual samples. A Mann–Whitney U test was performed to determine differences between the two groups (∗∗∗∗*P* ≤ 0.0001). **B**: Percentages (%) of children testing seropositive (*y*-axis) for anti-PGL-I IgM stratified by helminth infection status (*x*-axis). Anti-PGL-I units ≥1 (dotted line) were considered seropositive. Chi-squared tests were performed to test for differences in percentages between the two groups (∗∗∗*P* ≤ 0.001). IgM: immunoglobulin M; PGL-I: phenolic glycolipid-I.

In helminth-infected children, median anti-PGL-I IgM levels were significantly higher (Fig. 4A; Mann–Whitney: *P* < 0.0001). Among these children (n = 170), 22.4% (n = 38) tested seropositive for anti-PGL-I IgM compared to only 8.8% (n = 38 of 431) in those who were not helminth-infected (Fig. 4B; Chi-squared: *P* < 0.001).

## Discussion

This serosurvey contributes to the growing body of research assessing anti-PGL-I seroprevalence among children^28^, ^29^, ^30^ as an indicator of *M. leprae* transmission, building on previous studies from other endemic regions while providing new data from Indonesia using a field-friendly, rapid test. The study also explores associations with SES and helminth infection. Our study reveals a significant burden of (past or present) *M. leprae* infection among school-aged children in Indonesia, particularly in rural and low-SES settings. The overall seroprevalence of 12.2% in children is in line with previously reported seroprevalence in leprosy endemic areas using our test^12^^,^^13^ and underscores that transmission is ongoing despite elimination efforts. Markedly higher seroprevalence was found in rural areas such as Southwest Sumba (31.0%) and Pangkajene (11.2%) compared to urban areas like Makassar city (3.1%) and North Jakarta (3.8%). The mechanisms underlying higher *M. leprae* infection rates in rural settings remain incompletely understood. Transmission is thought to occur primarily from MB cases to susceptible individuals. However, several factors may contribute to higher seroprevalence in rural areas, including delayed diagnosis of index cases,^17^ limited access to healthcare and poor sanitation.^31^ The markedly higher prevalence of helminth infections in these areas may further increase susceptibility through immunomodulation.^32^ Recent studies have also suggested the potential role of soil and water as reservoirs of *M. leprae*,^33^ although more research is needed. While these hypotheses do not provide definitive explanations, they highlight the complex interplay of environmental, socioeconomic, and host factors that likely drive ongoing transmission in rural Indonesia.

Children attending low-SES schools exhibited seropositivity more frequently with significantly higher anti-PGL-I IgM levels than their high-SES counterparts, indicating higher transmission rates in the population where these groups originate from. Moreover, our findings further support the association between low-SES and increased exposure to *M. leprae*, highlighting that poverty-related factors indeed may contribute to heightened vulnerability to leprosy.^7^ This trend correlates with higher rates of undernutrition among low-SES children, supporting earlier findings that malnutrition impairs immune function and increases susceptibility to infection.^34^ In relation to this, Feenstra et al. reported a rise in leprosy cases following a period of famine in Bangladesh, further underscoring the role of nutritional stress in amplifying disease vulnerability.^35^ These data emphasize the need for holistic public health strategies that address both infectious diseases and nutritional deficiencies.

Our observation that female children exhibited significantly higher anti-PGL-I IgM levels than males is notable, given that males are generally reported to be more often affected by leprosy.^36^ However, leprosy in women is probably underreported due to sociocultural factors as well as stigma^36^ and elevated anti-PGL-I IgM levels among females have been documented previously.^29^^,^^30^ Nonetheless, the underlying reasons for this finding remain unclear and warrant further investigation.

Our findings indicate a clear association between helminth infection and elevated anti-PGL-I IgM levels. Seropositivity was 2.5 times higher among helminth-infected children (22.4%) compared to uninfected peers (8.8%). This reinforces the hypothesis that helminth-induced immunomodulation may increase susceptibility to *M. leprae* or delayed clearance of the pathogen.^8^^,^^9^^,^^32^ Given this correlation, there is a strong rationale for incorporating helminth prophylaxis as part of integrated public health strategies in regions with concurrent burdens of leprosy and STH. Improvements in sanitation may also contribute by lowering helminth prevalence, providing an additional mechanism for reductions in leprosy incidence. However, the introduction of helminth prophylaxis in leprosy-endemic settings must be approached with caution. Helminths are known to modulate host immune responses toward a Th2-biased profile, which may partially suppress Th1-type responses that are critical in leprosy control.^8^ Sudden removal of helminth-induced immunoregulation may theoretically unmask subclinical leprosy or precipitate immunological reactions, such as reversal reactions (Type 1) or erythema nodosum leprosum (Type 2).^8^ Therefore, any integration of deworming programs into leprosy control efforts warrants careful monitoring, along with clinical training to recognize and manage reactions effectively.

Southwest Sumba emerged in our study as the site with a striking seroprevalence of 31.0% among school-aged children, indicating high transmission. Although for the three other sites there seemed to be an association between the measured seroprevalence and NCDR, for Sumba seroprevalence in children did not align with the reported NCDR for the area. However, this could be due to the lack of proper leprosy health care programs in that area as a recent study reported that between 2020 and 2024, 60 individuals were diagnosed with leprosy (80% MB and 20% PB) in Southwest Sumba and suggested significant underreporting of cases.^17^ According to the same publication, the median interval between symptom onset and diagnosis was 36 months, and 33% of patients already had visible disabilities at diagnosis. These delays in diagnosis and treatment resulted from limited access to specialized dermatological care, with the nearest dermatologist located more than 24 h away by boat. Given the intensity of transmission in Southwest Sumba, targeted public health interventions, such as active case finding, and contact tracing including the provision of single-dose rifampicin as post-exposure prophylaxis (PEP) are urgently needed.^11^ With the baseline data provided in this study, using serosurveillance, Sumba could serve as a sentinel site for evaluating the effects of integrated control strategies. Moreover, the findings of this study underscore the utility of serosurveys among children in detecting otherwise hidden transmission.

While this study was primarily descriptive, future studies incorporating multivariate regression analyses are needed to disentangle the independent effects of SES, helminth infection, and nutrition on *M. leprae* infection. Moreover, our study is exploratory and not intended to be nationally representative. Districts and schools were purposively selected and generalizations beyond the sampled districts are limited. Nonetheless, this study clearly demonstrated the operational feasibility and utility of the PGL-I QURapid in large-scale, field-based serosurveillance efforts. Preparations for a larger-scale investigation are currently underway in other regions of Indonesia. The performance of the PGL-I QURapid with FSB allows for efficient deployment in the field, providing reliable data that can inform risk mapping and help targeting of interventions. The test enabled the identification of seropositive children without clinical symptoms, suggesting a potential role in identifying hidden transmission hotspots.

This study confirms ongoing *M. leprae* transmission in Indonesia, with particularly high seroprevalence among children in rural areas like Sumba. By detecting past or present infection, the PGL-I QURapid reveals transmission and offers a promising tool for both targeted prophylaxis guidance and post-intervention surveillance. Our findings support integrating serological surveillance into national leprosy programs, especially in low-SES and high endemic settings as well as areas thought to near or having reached elimination. Addressing factors like helminth infection and undernutrition alongside leprosy control may improve control effort outcomes. Monitoring child seroprevalence as a measure for recent transmission, as recommended by WHO, is key to tracking elimination progress and refining strategies.

## Contributors

Conceptualisation: LP, AG.

Data curation: LP, MM, GW, DE, HK, SW, SA, MMMK.

Formal analysis: LP, AG.

Funding acquisition: MMMK, AG.

Investigation: LP, MM, GW, DE, HK, SW, SA, MMMK, PC, AG.

Methodology: LP, AG.

Resources: SA, MMMK, PC, AG.

Supervision: AG.

Visualization: LP, AG.

Writing - original draft: LP, AG.

Writing - review & editing: LP, MM, GW, DE, HK, SW, SA, MMMK, PC, AG.

## Data sharing statement

The datasets used and/or analyzed during the current study are available from the corresponding author upon reasonable request.

## Declaration of interests

The authors declare that they have no conflict of interest.

## Appendix A Supplementary data

Supplementary Figs. S1–S3 and Table S1

Translated Abstract
