# Supplementary material for: Seroprevalence of Mycobacterium lepraeantibodies among school children in Indonesia in 2023: a cross-sectional study
Source: Lancet Reg Health Southeast Asia. 2026 May 27;49:100779. doi: 10.1016/j.lansea.2026.100779 (PMC13235328; doi:10.1016/j.lansea.2026.100779)
Supplement: Supplementary Figs. S1–S3 and Table S1 [file mmc1.docx]

**
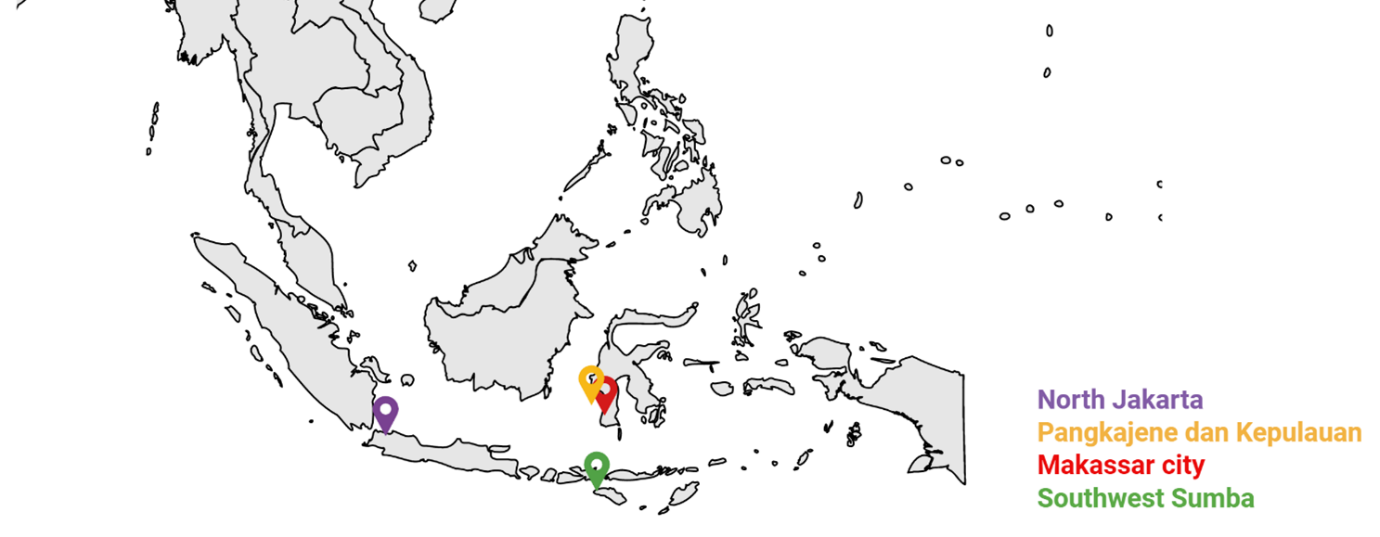
**

**Supplementary figure 1. Map of Indonesia showing the geographic distribution of study sites.**Map of Indonesia showing the four study locations where samples were collected from 637 children aged 6-15 years. Sites included schools in urban and rural areas in Sulawesi (Pangkajene: dark yellow; Makassar city: red), Java (North Jakarta: purple), and Sumba (Southwest Sumba: green). This figure was created using Biorender.

**
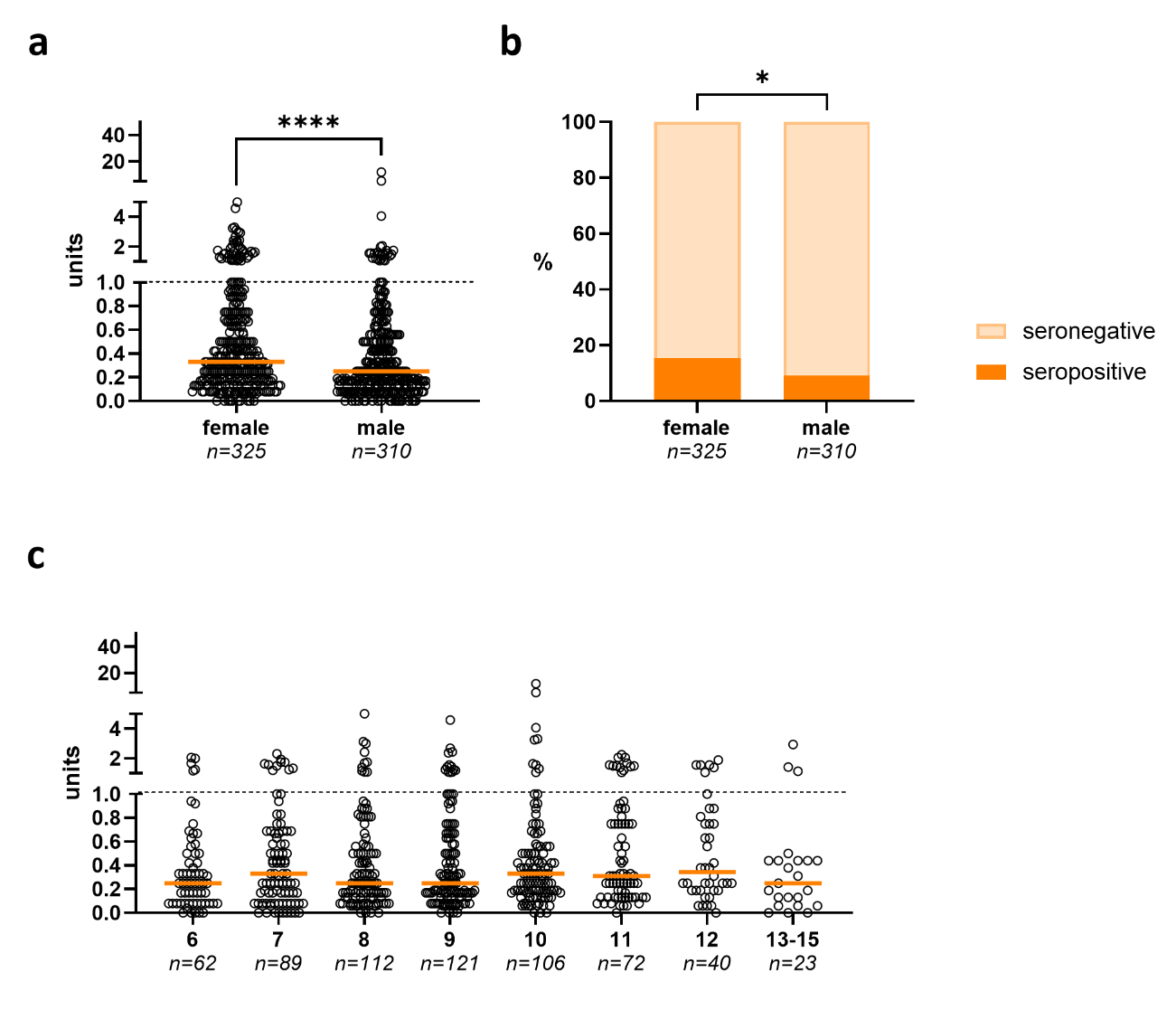
**

**Supplementary figure 2. Anti-PGL-I IgM in children stratified by gender and age.
A**: Anti-PGL-I IgM (in units; *y*-axis) of female and male children (*x*-axis). A Mann-Whitney U test was performed to determine differences between the two groups (**** *P ≤* 0.0001). Anti-PGL-I units ≥ 1 (dotted line) were considered seropositive. Two individuals were excluded from the analysis due to missing information on gender.  **B**: Percentages (%) of children testing seropositive (*y*-axis) for anti-PGL-I IgM stratified by gender (*x*-axis). Chi-squared tests were performed to test for differences in percentages between the two groups (* *P* ≤ 0.05).
**C**: Anti-PGL-I IgM (in units; *y*-axis) per age group (*x*-axis). A Kruskal-Wallis test with correction for multiple testing was performed to determine differences between the groups. Twelve participants were excluded from the analysis due to missing age information. Ages 13 to 15 were grouped together because of the low number of participants in these age categories. Dots (open circles) represent anti-PGL-I units for individual samples. IgM: immunoglobulin M; PGL-I: phenolic glycolipid-I.

**
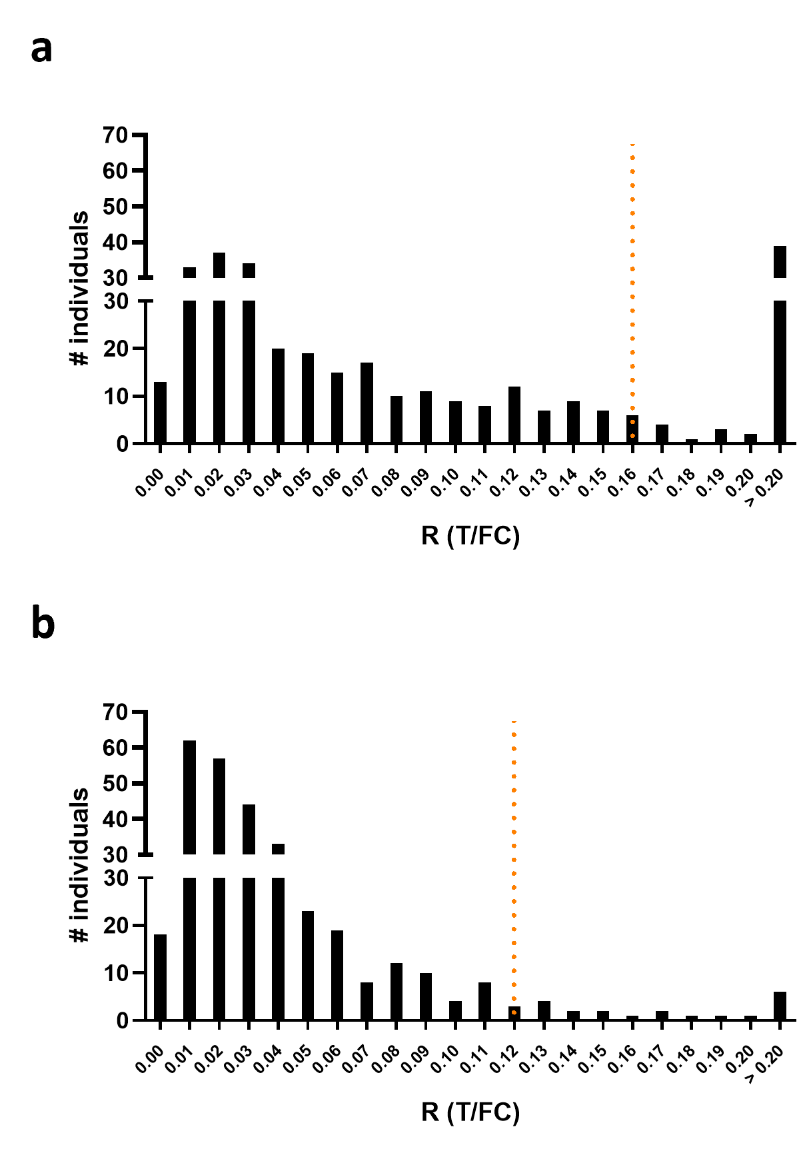
**

**Supplementary figure 3. Presence of anti-PGL-I IgM antibodies in plasma (A) and FSB (B) of schoolchildren in Indonesia.
A:** Histogram with a mode of 0.01 for R. The cut-off for positivity in plasma (R ≥ 0.16) for the UCP-LFA used in this study is indicated by the dotted line. **B:** Histogram with a mode of 0.01 for R. The cut-off for positivity in FSB (R ≥ 0.12) for the UCP-LFA used in this study is indicated by the dotted line.
FC: flow control line; IgM: immunoglobulin M; PGL-I: phenolic glycolipid I; R: ratio value; T: test line.

**Supplementary table 1. Leprosy new case detection rates (NCDR) for 2023 per district.**

| Island | Province | District | NCDR per 100,000 population |
| --- | --- | --- | --- |
| **Java** | Special Capital Region of Jakarta |  |  |
| **Sulawesi**  **Sumba** | South Sulawesi  East Nusa Tenggara | North Jakarta  Makassar city  Pangkajene  Southwest Sumba | 3.5  7.1  19.3  7.6 |

NCDR: new case detection rate.
