# Supplementary material for: Seroprevalence of Mycobacterium lepraeantibodies among school children in Indonesia in 2023: a cross-sectional study
Source: Lancet Reg Health Southeast Asia. 2026 May 27;49:100779. doi: 10.1016/j.lansea.2026.100779 (PMC13235328; doi:10.1016/j.lansea.2026.100779)
Supplement: Translated Abstract [file mmc2.docx]

*This translation in Bahasa was submitted by the authors and we reproduce it as supplied. It has not been peer reviewed. Our editorial processes have only been applied to the original abstract in English, which should serve as reference for this manuscript.*

**Ringkasan**

**Latar Belakang:** Kusta masih menjadi masalah kesehatan masyarakat yang signifikan di daerah endemik, termasuk wilayah di Indonesia. Penularan *Mycobacterium leprae (M. leprae)* masih terjadi, hal ini ditunjukkan dengan jumlah kasus kusta baru yang tetap stabil setiap tahun, dengan sekitar 5% di antaranya adalah kasus kusta anak. Untuk menghentikan penularan *M. leprae*, penting untuk mengidentifikasi dan mengobati sumber infeksi. Studi ini bertujuan menilai prevalensi serologi IgM anti fenolik glikolipid-I (PGL-I) *M. leprae* pada anak sekolah usia 6-15 tahun di daerah pedesaan dan perkotaan Indonesia, sebagai indikator penularan baru. Studi ini juga mempelajari hubungan antara seropositifitas dan jenis kelamin, status sosial ekonomi (SES), serta infeksi cacing usus.

**Metode:** Kami melakukan survei serologi potong lintang pada 637 anak (usia 6-15 tahun) dari sekolah di daerah perkotaan dan pedesaan di Sulawesi, Jawa, dan Sumba, Indonesia. PGL-I QURapid, sebuah tes lateral flow kuantitatif yang mudah digunakan di lapangan, digunakan untuk mendeteksi antibodi IgM anti-PGL-I dalam sampel darah jari dan plasma. Data sosioekonomi dan kesehatan, termasuk status infeksi cacing dan skor z indeks massa tubuh (z-BMI), juga dikumpulkan.

**Temuan:** Prevalensi serologis anti-PGL-I IgM pada anak-anak secara keseluruhan sebesar 12,2%, dengan prevalensi serologis yang secara signifikan lebih tinggi di daerah pedesaan (Sumba Barat Daya: 31,0%, Pangkajene: 11,2%) dibandingkan dengan daerah perkotaan (Jakarta Utara: 3,8%, Kota Makassar: 3,1%). Selain itu, seroprevalensi pada anak-anak dari sekolah dengan status ekonomi sosial (SES) rendah secara signifikan lebih tinggi (13,7%) dibandingkan dengan anak-anak dari sekolah dengan SES tinggi (4,1%, P = 0,007). Kehadiran infeksi cacing terkait dengan tingkat anti-PGL-I IgM yang secara signifikan lebih tinggi (P < 0,0001), dengan 22,4% anak yang terinfeksi cacing menunjukkan hasil seropositif dibandingkan dengan 8,8% anak yang tidak terinfeksi (P < 0,001).

**Interpretasi:** Temuan kami menunjukkan prevalensi seropositif anti-PGL-I IgM yang tinggi pada anak-anak di pedesaan, dengan SES rendah, dan terinfeksi cacing di Indonesia, menunjukkan tingkat infeksi M. leprae yang tinggi. Hal ini mencerminkan tingkat penularan yang lebih tinggi di populasi. Hasil ini menekankan perlunya strategi pengendalian penyakit terintegrasi yang menargetkan baik lepra maupun helminthiasis, terutama di daerah pedesaan dan ekonomi yang kurang berkembang. PGL-I QURapid terbukti menjadi alat yang efektif dalam survei serologi, mampu membantu identifikasi daerah dengan tingkat penularan M. leprae yang tinggi (seperti Sumba) dan mendukung implementasi intervensi terarah di daerah endemik.

**Pendanaan:** Studi ini didanai oleh hibah dari Yayasan Q. M. Gastmann-Wichers (AG) dan hibah internal dari Universitas Katolik Atma Jaya Indonesia (MMMK).
